# Diversity and Antimicrobial Potential of Predatory Bacteria from the Peruvian Coastline

**DOI:** 10.3390/md15100308

**Published:** 2017-10-12

**Authors:** Luis Linares-Otoya, Virginia Linares-Otoya, Lizbeth Armas-Mantilla, Cyntia Blanco-Olano, Max Crüsemann, Mayar L. Ganoza-Yupanqui, Julio Campos-Florian, Gabriele M. König, Till F. Schäberle

**Affiliations:** 1 Institute for Insect Biotechnology, Justus Liebig University of Giessen, 5392 Giessen, Germany; luis.lioto@hotmail.com; 2 Institute for Pharmaceutical Biology, University of Bonn, 3115 Bonn, Germany; mcruesem@uni-bonn.de (M.C.); g.koenig@uni-bonn.de (G.M.K.); 3 Department of Pharmacology, Faculty of Pharmacy and Biochemistry, National University of Trujillo, 13011 Trujillo, Peru; mvlinareso@hotmail.com (V.L.-O.); lizfa_2712@hotmail.com (L.A.-M.); cyntia_0212@outlook.com.pe (C.B.-O.); mganoza@unitru.edu.pe (M.L.G.-Y.); jcamposf@unitru.edu.pe (J.C.-F.); 4 German Centre for Infection Research (DZIF) Partner Site Bonn/Cologne, Bonn 53115, Germany; 5 Research Centre for Sustainable Development Uku Pacha, 13011 Uku Pacha, Peru

**Keywords:** antibiotics, dereplication, microbiome, natural products, predatory bacteria

## Abstract

The microbiome of three different sites at the Peruvian Pacific coast was analyzed, revealing a lower bacterial biodiversity at Isla Foca than at Paracas and Manglares, with 89 bacterial genera identified, as compared to 195 and 173 genera, respectively. Only 47 of the bacterial genera identified were common to all three sites. In order to obtain promising strains for the putative production of novel antimicrobials, predatory bacteria were isolated from these sampling sites, using two different bait organisms. Even though the proportion of predatory bacteria was only around 0.5% in the here investigated environmental microbiomes, by this approach in total 138 bacterial strains were isolated as axenic culture. 25% of strains showed antibacterial activity, thereby nine revealed activity against clinically relevant methicillin resistant *Staphylococcus aureus* (MRSA) and three against enterohemorrhagic *Escherichia coli* (EHEC) strains. Phylogeny and physiological characteristics of the active strains were investigated. First insights into the chemical basis of the antibacterial activity indicated the biosynthetic production of the known compounds ariakemicin, kocurin, naphthyridinomycin, pumilacidins, resistomycin, and surfactin. However, most compounds remained elusive until now. Hence, the obtained results implicate that the microbiome present at the various habitats at the Peruvian coastline is a promising source for heterotrophic bacterial strains showing high potential for the biotechnological production of antibiotics.

## 1. Introduction

Bacteria have developed diverse strategies and remarkable metabolic capabilities to survive and to colonize harsh environments, such as marine habitats [1]. Certain bacteria apply the strategy to live in association with larger organisms, e.g., sponges or sea slugs, while others have developed a more independent lifestyle [2,3]. The latter group consists of more autonomous bacteria that in most cases possess larger genomes, which provide them with a larger metabolic repertoire enabling them: (i) to get access to scarce nutrients, and/or (ii) to produce bioactive specialized metabolites to protect themselves from predators or even to play this role [4].

Predatory behavior is widely distributed among bacteria and can be found in several phyla, e.g., in Actinobacteria [5], Bacteroidetes [6], Chloroflexi [7], and Proteobacteria [8]. However, the strategies that these microorganisms apply to foster a predatory lifestyle are variable. For a *Cytophaga* species, it was reported that it does epibiotic predation [9]. This means that it attaches to the prey, i.e., phytoplankton, surface, followed by degradation and assimilation of the cell. The Proteobacteria *Bdellovibrio* spp. are obligatory predatory bacteria, which apply a periplasmatic type of predation [8]. They grow epibiotically, whereby the predator cells are attached to the cell envelope of the prey. They are dividing in a binary manner by penetrating the prey’s periplasm to generate a number of progeny cells [10]. Another most interesting predatory behavior was denominated as the ‘wolfpack strategy’ [11]. Here, a predatory attack is performed by using sophisticated communication systems, since it involves a coordinated attack by many bacterial cells, which have the ability to glide on surfaces. A famous example are the Myxobacteria, e.g., the marine *Enhygromyxa salina* [3] and *Cystobacter* sp. [12], well-known proliferative producers of antibiotically active metabolites [13,14]. Further examples come from the Gammaproteobacteria, e.g., *Lysobacter* spp. [15], the Bacteroidetes, e.g., *Rapidithrix thailandica* [16], *Porifericola rhodea* [17], and the Chloroflexi, e.g., *Herpetosiphon* spp. [7]. As mentioned before, a characteristic feature of these taxa is their large genome size with a striking high number of biosynthetic gene clusters (BGCs) putatively coding for specialized metabolites. It is assumed that the biosynthesis of antibiotics plays an important role in their predatory lifestyle [4].

The assumption that these bacteria use antibiotics to weaken or even kill their prey [4] implements the great potential of these organisms as source for novel bioactive compounds. The goal of this project was to identify new bacterial species, which possess the potential to biosynthesize antibiotically active natural products. Therefore, the Peruvian coastline was selected as sampling area, since it represents a biodiverse ecosystem rich in endemic species [18]. It is of interest, if the grade of biodiversity, usually judged by macroorganisms, can also be transferred to microorganisms. Thereby, in this study, the focus is on predatory bacteria. Hence, after obtaining culture-independent insights into the bacterial diversity, a culture-dependent approach using the predatory behavior of the target organisms was applied to isolate promising strains that could potentially produce new antibiotics.

## 2. Results

### 2.1. Coastal Microbiome

**Figure 1:**
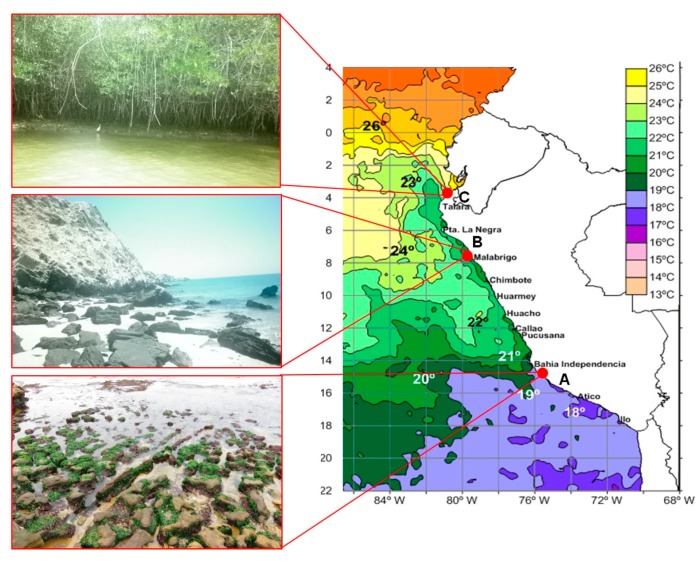
Sample collection sites. The sites are indicated by a red dot at the map. The water temperature (annual average) is given. A: Paracas (18 °C), open beach with presence of halophilic algae and remains of mollusk shells in the collected sediment; B: Isla Foca (22 °C), island located at the confluence of the Humboldt stream and the Caribbean. Presence of coralliferous formations and endemic species in the sampled soil; C: Manglares (26 °C), mangrove ecosystem located at the mouth of the river Tumbes in the Pacific Ocean. Collected samples consisted of mud, located at the base of mangroves.

In order to get an insight into the bacterial diversity present at the three coastal sites, the metagenome of the soil samples was analyzed. The sampling sites represent three marine ecosystems with different seawater temperature (Figure 1).

**Figure 2:**
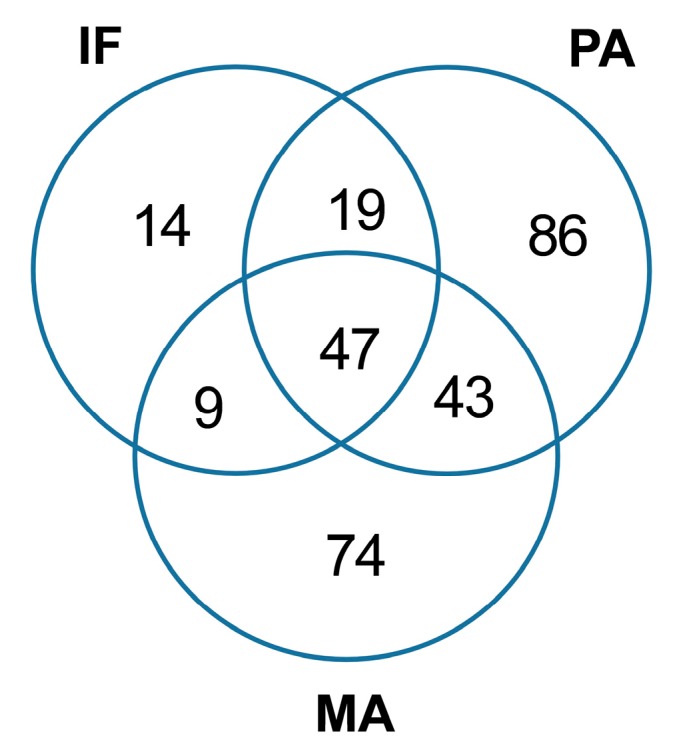
Venn diagram showing site specific and shared bacterial genera. The three sites are Isla Foca (IF), Paracas (PA), and Manglares (MA).

Paracas is located in the southern coastline of Peru, clearly dominated by the Humboldt Current that brings a cold-water column from southern latitudes. Isla Foca represents the encounter point between the Caribbean and Humboldt Current. Manglares instead, is a mangrove ecosystem, located in the estuary of the Tumbes River, close to the Pacific Ocean. Analysis of the 16S rRNA data obtained by next generation sequencing showed that a less diverse microbiome was found at Isla Foca, IF (213,991 reads) where 89 bacterial genera were found, as compared to 195 genera at Paracas, PA (161,958 reads) and 173 genera at Manglares, MA (163,056 reads). 47 bacterial genera, equivalent to one-half of the IF genera and about one fourth of the PA and MA genera, were found at all of the sites (Figure 2). Approximately half of the genera present in the samples of PA and MA were specific for the respective site, while at the IF site only one sixth of all bacteria could be contributed to specific genera.

**Figure 3:**
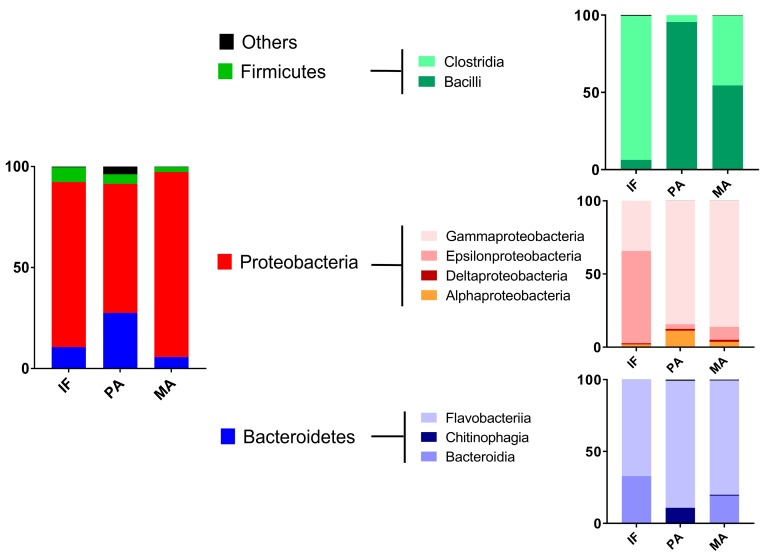
Relative abundance of the bacterial taxa in the metagenomic analysis of the coastal samples.

Regarding the phylum level, the sites show a comparable pattern, with Proteobacteria being the most dominant representative (81.45% IF, 63.22% PA, 91.21% MA), followed by Bacteroidetes (10.60% IF, 27.21% PA, 5.69% MA). The remaining portion consists of Firmicutes (7.28% IF, 4.63% PA, 2.50% MA) and in the case of PA 4.94% others (Figure 3).

At the lower taxa level of classes the differences between sites were even more pronounced. Gammaproteobacteria dominated the Proteobacteria clade for PA and MA, while at the IF site the Epsilonproteobacteria were the most abundant class. The latter class was almost completely built by Campylobacterales, which summed up to 98.3 ± 2.33%. In the Gammaproteobacteria phylum the Alteromonadales (IF: 18.81%, PA: 33.18%, MA: 54.55%) and Oceanospirillales (IF: 16.4%, PA: 32.72%, MA: 18.28%) represented the most abundant orders, with the exception of IF where a high abundance of the Vibrionales (36.97%) and pseudomonads (25.54%) was observed. The most abundant class within the Bacteroidetes phylum was at all of the sites Flavobacteria (78.03 ± 10.36). Therein, the Flavobacteriaceae accounted for 85.69 ± 12.43%. At IF and MA the Bacteroidia class accounted nearly for the complete rest of the bacterial clades in the phylum Bacteroidetes. At PA instead, the Chitinophagia class represented the remaining bacteria. Other orders within the Bacteroidetes phylum were <1%. The Firmicutes split mainly into Clostridiales and Bacillales, other bacteria had a proportion <1%. However, the ratio of Bacillales to Clostridiales was 1:14.9 in PA, 21.8:1 in IF and 1.2:1 in MA. Hence, only in the mangrove coastal environment of Manglares both classes were present in similar proportions.

Beside the general insights into the bacterial diversity present at the Peruvian coastline the potential for detection of antibiotically active metabolites should be investigated. Therefore, it was first planned to target Myxobacteria, as reported proliferative secondary metabolite producers [13]. However, only a neglectable amount of the reads accounted for myxobacterial species, i.e., only 1 read (equivalent to 0.0002%) could be contributed to *Myxococcus xanthus*. However, the rationale to isolate predatory bacteria, since it can be assumed that these organisms produce natural products with antibacterial activity to weaken or even kill their prey [19], could still be followed. A database of predatory bacteria was constructed based on literature (Table S4). Screening the obtained metagenomic data for the presence of reported predatory bacterial taxa, yielded in 0.50 ± 0.44% of the total number of reads (Table 1). At PA and MA, the abundance of the targeted bacteria was 0.67% and 0.85% of total bacteria, respectively. In contrast, IF showed just 19 reads (0.009%).

The most abundant clade of predatory bacteria particularly seen at MA corresponds to *Bdellovibrio*-like organisms (BALOs group), mostly represented by Bdellovibrionales (mainly *Bdellovibrio* and *Bacteriovorax*) (Table 1). The BALOs are characterized by their parasitic lifestyle on other Gram-negative bacteria and represented 86.07% of the predatory bacteria in the sample, while it summed up to 26.31% and 37.88% at IF and PA, respectively. Most of the predatory bacteria at IF (73.7%) and PA (62.02%) belonged to the taxon Bacteroidetes, while only 13.8% were found at MA. In minute proportions, the myxobacterium *Myxococcus xanthus* (specific for PA) and the cyanobacterium *Vampirovibrio* sp. (specific for MA) were observed.

### 2.2. Isolation of Antibiotic Producing Predatory Bacteria

**Figure 4:**
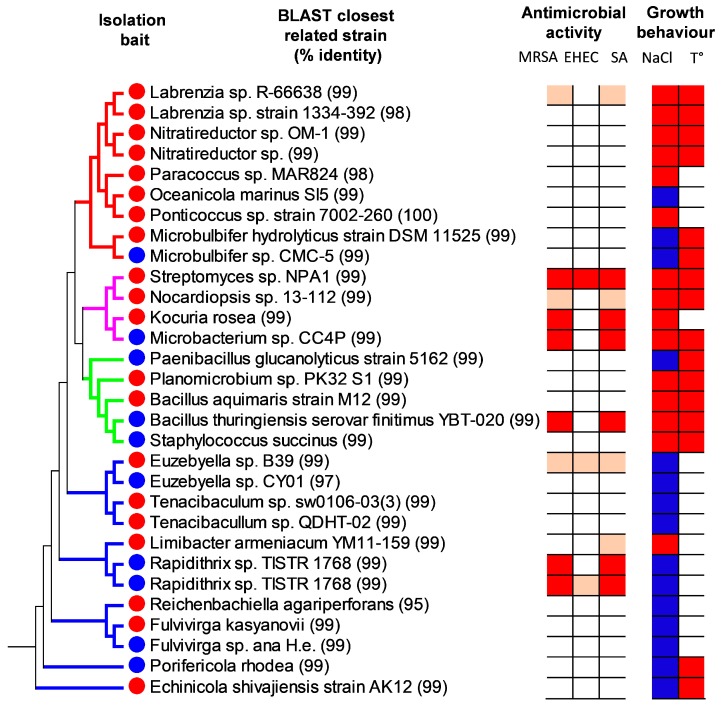
Phylogenetic tree of antimicrobially active isolates. All of the strains indicated here showed activity against *Arthrobacter psychrolactophilus*. The color code of the tree branches indicates Proteobacteria (red), Actinobacteria (green), Firmicutes (purple), and Bacteroidetes (blue). The column “Isolation bait” indicates if the strain was isolated using *E. coli* (red), or *P. inhibens* (blue) as prey organism. Antimicrobial activity is given against methicillin-resistant *Staphylococcus aureus* (MRSA), enterohemorragic *E. coli* (EHEC), and methicillin-sensitive *S. aureus* (SA). Dark and light red indicate strong and moderate antimicrobial activity, respectively; white indicates that no activity was observed. Physiological properties of the strains are indicated on the right side. The NaCl column indicates if the strain is able to grow optimally with low NaCl concentration (<1%, red) or if it needs higher amounts (>1.5%, blue). The T° column indicates which strains were able to grow at an elevated temperature (40 °C). The 16S rRNA sequences of the strains have been deposited at GenBank with the accession numbers MF796603-MF796631 and MF620093.

In the metagenomic analysis, it was shown that only about 0.5% of the bacteria present in the environmental samples correspond to known predatory bacteria. To get a hand on these underrepresented strains, we used the bait-streak isolation technique (see Material and Methods). Two different bacterial strains were used as bait organisms, i.e., *Escherichia coli* XL1 Blue and the marine Alphaproteobacterium *Pheobacter inhibens* DSM17395. By this approach, 138 bacterial strains were isolated. Thereby, 105 strains were isolated from *E. coli* bait plates and 33 were obtained using *P. inhibens* as bait. It was noticed that the observable growth was significantly slower if *P. inhibens* was used as bait (14–21 days), than if *E. coli* was used (6–10 days). All of the obtained isolates were screened for their antibiotic activity. Liquid cultures were extracted with ethyl acetate and the resulting crude extracts were initially tested against Gram-positive *Arthrobacter psychrolactophilus* and Gram-negative *E. coli* strains. As a result 35 bacterial strains (equivalent to 25%) showed activity against *A. psychrolactophilus* and four strains (3%) against *E. coli* (Figure 4, Table S1).Thereby, ten of the active strains had been isolated using *P. inhibens*, and 25 strains using *E. coli* as prey organism. These strains, showing antibiotic activity in the first screening round, were further tested against clinical isolates, i.e., MRSA and EHEC strains. Now, nine bacterial strains showed activity against MRSA. These bacterial strains were members of the genera *Bacillus* (1), *Euzebyella* (1), *Kocuria* (1), *Labrenzia* (1), *Microbacterium* (1), *Nocardiopsis* (1), *Rapidithrix* (2), and *Streptomyces* (1). Three strains also exhibited antimicrobial activity against EHEC (equivalent to 1.4% of the isolated 138 strains). These strains showed highest homology to *Rapidithrix thailandica* TISTR1768 (99% identity on DNA level), *Streptomyces* sp. NPA1 (99%), and *Euzebyella* sp. B39 (99%).

### 2.3. Phylogenetic Analysis

From the originally isolated 35 strains showing antimicrobial activity, 30 could be retained in vitro, since five strains stopped growing under our culture conditions. The in vitro culturable strains were identified by 16S rRNA gene sequencing. The NCBI database was used to determine the closest relatives by using BLASTn and a phylogenetic tree was constructed using the minimal evolution method. Most of the active strains (12 strains) belong to the Bacteroidetes phylum; therein especially the Cytophagales family is pronounced (8 strains) (Figure 4). This fact clearly indicates that this clade of predatory bacteria was highly enriched by the isolation method used in this work, since it represented only 0.1% of the total bacterial counts in the metagenomic analysis. The enrichment of this clade was even more pronounced when *P. inhibens* was used as bait (5 out of 10 strains).

In the Proteobacteria phylum, the Alphaproteobacteria (7 strains) were enriched as compared to the Gammaproteobacteria (2 strains). This differs to the relative abundance observed in the metagenomic data where the latter represent about 80% of the Proteobacteria. The strains isolated from this clade, however, were not described to have a predatory behavior, but could be isolated by the method used. Thereby, Alphaproteobacteria were only isolated by using *E. coli* as bait (7 out of 25 strains as compared to 0 out of 10 strains by using *P. inhibens*).

Apart from the Bacteroidetes and Proteobacteria, the remaining 9 strains were divided between the Firmicutes and Actinobacteria phylum. It was observed that *E. coli* used as a bait during isolation tended to enrich Actinobacteria (3 strains were isolated using *E. coli* and only 1 strain using *P. inhibens*) while *P. inhibens* bait enriched Firmicutes (4 strains were isolated using *P. inhibens* and only 1 strain using *E. coli*).

### 2.4. Physiological Properties of the Isolated Bacteria

All of the strains grew optimally when the NaCl concentration in the medium was around 2%, and nearly all of the strains were able to grow in the NaCl range from 0.5 to 3.89% (Table S2). However, four strains from the Bacteroidetes (*Echinicola* sp. and *Tenacibaculum* spp.) and Proteobacteria (*Oceanicola* sp.) clades seemed to be obligatory marine, since they stopped growing when the NaCl concentration in the media was decreased below 1%. The *Oceanicola* sp. even showed the best growth rate around 4% and was unable to grow under 2% NaCl. Most of the bacterial strains grew optimally to around 30 °C (Table S3). However, several strains were able to grow at higher temperatures of 45 °C. These were mainly the Actinobacteria (with the exception of *Kocuria* sp.) and Firmicutes. In addition, Proteobacterial strains, e.g., *Labrenzia* and *Nitratireductor*, and few Bacteroidetes, e.g., *Porifericola* and *Echinicola*, showed a tolerance to higher temperatures.

### 2.5. Identification of Known Compounds—Dereplication

In order to get a better picture of the metabolome of the isolated bacteria and to identify known antibiotic compounds produced, a Global Natural Product Social Molecular Networking (GNPS) analysis was performed [20]. In addition, a literature database search was done, in order to dereplicate additional compounds.

The ethyl acetate crude extracts were analyzed by HPLC-MS/MS and the GNPS server enabled us to identify several reported compounds with antimicrobial activity. This analysis showed that many compounds are exclusively biosynthesized by a particular strain. When comparing the two isolated *Rapidithrix* strains, which share 99% identity of their 16S rRNA gene, revealed that only one strain, i.e., *Rapidithrix thailandica* s80 produced the hybrid peptide-polyketides ariakemicin A and B. The other strain, i.e., *Rapidithrix thailandica* s68, instead, showed different metabolites that were not found in the former one. Further, we were able to identify several already described antibiotics using mass-based analysis: Kocurin was identified in extracts from *Kocuria rosea* s17 [21]; naphthyridinomycin and resistomycin in *Streptomyces* sp. s120; and, the pumilacidins C and E as well as surfactin in *Paenibacillus glucanolyticus* s102b (see Supplementary Information).

A first draft of the genome sequence of *Streptomyces* sp. s120 (unpublished) was analyzed using antiSMASH [22] and biosynthetic gene clusters showing similarities to reported naphthyridinomycin and resistomycin clusters could be detected (Table S5). However, for most of the antibiotically active extracts the responsible molecule(s) could not be identified yet.

## 3. Discussion

Predatory bacteria play an important role in marine ecosystems. It was demonstrated that members of the BALOs, i.e., *Bdellovibrio bacteriovorus* and *Bacteriovorax stolpii*, as well as *Micavibrio aeruginosavorus*-like predators are key players in controlling the abundance of several bacteria. All are epibiotic bacteria, whereby the *M. aeruginosavorus*-like species do not invade the periplasmatic space of their prey. Together they are important modulators of *Vibrio* populations in seawater and oysters [23]. In the here reported sample set, the BALOs represented about 70% of the total predatory bacteria present. Notably, this group was more abundant at the MA and PA sites. It is however difficult to link this to the abundance of a particular prey taxa, since the host specificity from these strains can be variable and differs from one strain to another [24]. However, it can be assumed that their prey was not so abundant at the IF site, at least at the time the samples were taken, since for these predatory taxa only five reads were detected. Instead, Epsilonproteobacteria and Clostridiales were more dominant at IF, indicating a more anaerobic and/or sulfur rich environment, which does not represent a favorable habitat for the before mentioned strictly aerobic taxa [25]. The overall percentage of 0.50 ± 0.44% of predatory bacteria in the environmental samples corresponds to reported values. In marine aquaculture systems the observed range was 0.13–1.4% [26].

These previously mentioned taxa that require live prey as their growth substrate represented the majority of predatory bacteria found in the investigated sediment samples. However, in regard of proliferative producers of biologically active metabolites, the facultative predators represent the more promising species, e.g., Myxobacteria, Cytophagales and certain Flavobacteria. In order to get nutrients by predation, these bacteria make use of their gliding motility and the biosynthesis of a whole repertoire of bioactive specialized metabolites. These metabolites can be used for intra- and interspecies communication, as well as for chemical warfare [4]. Remarkably, such biologically active metabolites are of high interest for the pharmaceutical industry, due to their potential utility in modern medicine and biomedical research [27]. As here exemplified by two phylogenetically distinct predatory bacteria, i.e., *Myxococcus* species (Myxobacteria) and the *Herpetosiphon* species (Chloroflexi) [4]. Both possess large genomes with an overrepresented number of BGCs, putatively involved in the biosynthesis of specialized metabolites. Corresponding molecules, showing promising activities, were already isolated, e.g., the antibiotics myxovirescins and gulmirecins from *Myxococcus*, as well as siphonazole and auriculamide from *Herpetosiphon* [4].

Even though the share of predatory species in the environmental microbiome is low, they can be isolated and mostly adapted to grow under laboratory conditions. The isolation based on the phenotype is an important selection criterion, since also closely related strains might be non-predatory. Hence, starting with culture-independent screening approaches is difficult. It was reported that the predatory bacterium, *Ensifer adhaerens*, is very closely related to a non-predatory strain, which rendered 16S rRNA gene-based phylogenetic probes incapable of distinguishing predatory from non-predatory organisms [28]. In addition, predatory lifestyle can be encountered in various taxa with different phylogeny and physiology. Using two different Proteobacterial strains as bait, i.e., the Gammaproteobacterium *E. coli* and the Alphaproteobacterium *P. inhibens* resulted in the isolation of targeted bacteria that initially were not even identified by the metagenomic analysis. In that way, two *Rapidithrix* species and one *Porifericola* species were isolated, described as predatory bacteria before [16,17]. Hence, the power of the isolation method is demonstrated, although it became clear that the selection of the prey organism has a strong influence onto the bacterial strains isolated. Particular taxa were isolated using a given prey organisms, e.g., there was a complete lack of Alphaproteobacterial isolates when *P. inhibens* was used as bait. This result is in accordance with previous work performed to assess the prey specificity of several *Tenacibaculum* strains [29]. They reported clear differences in prey preferences even at the intra-genus level. This might also explain why during this work several strains were initially isolated, but after several transfers, they stopped growing in vitro, i.e., *Brumimicrobium*, *Fulvivirga*, and *Saprospira* species. For future bioprospecting projects, the use of several taxa as bait organisms should allow to diversify and/or enrich certain bacteria, thereby increasing the chance for the isolation of novel strains. In addition, the methods to isolate such low abundant species like the predatory bacteria should be developed further. No myxobacterial species could be isolated, even though a *M. xanthus* read was detected by metagenomics. It is assumed that Myxobacteria, which are detected using culture-independent methods, but cannot be cultivated from an environmental sample, are present as a vegetative cell form and not as dry-resistant myxospores [30]. If diffusion chambers like the iChip, which was successfully applied in the isolation of the teixobactin-producing bacterium *Eleftheria terrae* [31], can be successfully applied to isolate social bacteria, which hunt as wolf packs, must be evaluated in the future.

Interestingly, by the approach applied in the project strains belonging to the Actinobacteria and Bacillales taxa were isolated, which had not been reported before as predatory bacteria. However, strains of these taxa are well known as proliferative producers of specialized metabolites. This means that they developed different features to outcompete other bacteria in the highly competitive environment of the ocean to access nutrients. In fact, about 80% of natural products that are used in human medicine are derived from one genus, i.e., *Streptomyces* [32]. There are some possible explanations why such strains were isolated by our approach. It might be that they produce metabolites lysing the prey cells, and subsequently these metabolites can be used for growth, or they metabolize the agar present in the plates. Further, it has to be considered that in the environment and therewith in the samples used for cultivation, consortia of bacteria are present. Hence, predatory bacteria could initially release the nutrients from the prey, making them accessible for other strains, whereby we isolated the latter as axenic culture.

Even though Actinobacteria, especially Streptomycetes, are well investigated, and this group is still far from being completely exploited. Fostered by the progress in sequencing technologies, many genomes became available and using genome mining tools it can be clearly seen that many more BGCs coding for the synthesis of so far unknown metabolites are encoded in the genomes than compounds are identified. Therefore, such bacteria are still in the focus of natural product research. In this work, four Actinobacteria were isolated, one each from the genera *Kocuria*, *Microbacterium*, *Nocardiopsis* and *Streptomyces*, respectively. By the dereplication approach used, the thiopeptide kocurin was identified in the extract of *Kocuria rosea* s17. This potent anti MRSA antibiotic is produced by several members of the *Micrococcaceae* family. The activity profile of kocurin is comparable to linezolid, a drug in clinical use. In a in vivo mouse model infections with MRSA and VRE could be cured [33]. Coming back to the imbalance between BGCs identified in silico and known metabolites, kocurin is a special case, since it was the other way round. The compound was already known, but the biosynthesis remained unknown. Based on the identification of the compound in this project, we recently were able to identify and clone the corresponding BGC [21]. Moreover, by dereplication the polyketides resistomycin and naphthyridinomycin were identified in the crude extract of *Streptomyces* sp. S120.

Among the bacteria isolated in this project, the Cytophagales were highly enriched. For this phylogenetic clade the gliding behavior and in less degree epibiotic predation seem to be common features. Moreover, the strains belonging to this group seemed to be truly marine, since their growth was significantly reduced, once the NaCl level decreased below 1.5–2%. Three different *Fulvivirga* strains were initially isolated, however, only two could be maintained under the laboratory conditions used. From this genus, only one genome sequence is available in the NCBI database, i.e., *Fulvivirga imtechensis* (NZ_AMZN00000000.1). The genome has a size of approximately 7 mbps and a bioinformatic analysis using antiSMASH [22] revealed the presence of several BGCs that putatively code for specialized metabolites. This suggests *Fulvivirga* strains to be a promising target to continue research on the chemistry of this poorly investigated genus.

Other Bacteroidetes that could potentially offer novel antimicrobially active molecules are the Flavobacteria. In this work, two *Euzebyella* strains, showing 96% identity to each other, were isolated and showed activity against MRSA and *E. coli*. To the best of our knowledge, there is no previous report of antimicrobial compounds produced by this recently discovered genus [34]. Further research is planned to elucidate the chemical basis of the observed activities. The other predatory Flavobacterium isolated belonged to the genus *Tenacibaculum*. This taxa exhibits an epibiotic predation type [29]. Hence, this does not imply the production of specialized metabolites in the magnitude of the previously mentioned strains. This fact is also reflected in the much smaller genome sizes (3–3.5 mbps) of this genus.

However, there have been strains isolated belonging to the Cytophagales group for which antibiotic production was proven. Marine strains of the genus *Rapidithrix* or *Porifericola* were isolated with similar isolation techniques as the ones used here [16,17]. These strains showed complex gliding behavior and exhibited interesting antimicrobial activity. For *Rapidithrix* sp. HC35 the production of the antibiotic ariakemicin was reported [35], as well as several antibiotically active pyrrole derivative molecules [36,37]. In this work we isolated two antibiotically active *Rapidithrix* strains. For one of them the antimicrobial activity was attributed to ariakemicin production, while the other strain should produce different antibiotic compounds. Interestingly, the LC-MS/MS analysis revealed that the latter produced several compounds, which were not observed in the former. An intriguing fact is that other predatory bacteria, e.g., *Herpetosiphon* sp. B060 and *Sorangium* sp. (formerly reported as *Polyangium* sp.), produce specialized metabolites, which show structural similarities to the polyketide peptide hybrids ariakemicins [7,38]. Siphonazole, produced by *Herpetosiphon* sp. B060, and phenoxan, produced by *Sorangium* sp., have the benzoic acid derivative and the oxazole rings interlinked by unsaturated polyketide-peptide chain(s) in common. It was speculated that the production of related metabolites by phylogenetically distant taxa may imply that these natural products have the same molecular ancestor, diversified during evolution [35]. However, also more recent horizontal transfer events should be taken into account, since it was suggested that this could be the case for the peptide antibiotic althiomycin that was initially reported in *Streptomyces* and later in myxobacterial strains [4].

In summary, predatory bacteria play an important role in marine ecosystems and the physiological requirements for their lifestyle renders them a most promising source of bioactive compounds. Bioprospecting projects aiming to discover new producer strains of biologically active natural products have regained interest in the last years, given the notable development of other areas, such as improved analytical methods as well as the advances in sequencing technologies and in bioinformatics. These technologies complement the traditional screening efforts and provide us a more accurate look on the metabolic potential of these bacteria [20]. These new approaches for antibiotic discovery are particularly important given the current scenario, where the number of effective antibiotics is diminishing in an alarming rate and the panorama is not optimistic since, with a few exceptions, no major breakthrough discoveries similar to penicillin and aminoglycosides have been reported in the last 20 years [39]. Here, it could be shown that many strains possess antibacterial activities, whereby the chemical basis of this effect is unknown. Hence, these unexplored specialized metabolites possess likely novel chemical features. This in turn could contribute to the refilling of the antibiotics development pipeline. Further, it suggests using a higher number of prey organisms in future bioprospecting project, since this should diversify, or enrich particular taxa of bacteria to be isolated, thereby increasing the chance to isolate novel strains and compounds.

## 4. Materials and Methods

### 4.1. Collection of Samples and Isolation of Bacterial Strains

Intertidal soil samples containing organic material (0 to 0.5 cm deep) were collected from three different locations of the Peruvian coastline. The coordinates were: A: Paracas, S 13°51′44.8′′ W 76°16′11.7′′; B: Isla Foca, S 5°12′31.3′′ W 81°12′17.3′′; C: Manglares, S 3°25′33.8′′ W 80°16′28.6′′. These sampling areas were selected by taking the differences in seawater temperature into account, since variations in biodiversity can be expected in these habitats: Manglares (26 °C, northern coast), Isla Foca (22 °C, nor-central coast), and Paracas (18 °C, southern coast). The location of these areas is shown in Figure 1. The samples were transported in sterile falcon tubes to the Laboratory of Pharmacology of the National University of Trujillo. Bacteria isolation was based on the bacteriolytic properties of the expected strains. Therefore, *E. coli* XL1 Blue cells were incubated overnight in 50 mL LB (Luria Broth) medium at 37 °C, and *Pheobacter inhibens* DSM17395 cells were incubated in Marine Broth 2216 (Difco, Sparks, MD, USA) medium at 30 °C, respectively. Then, cells were collected by centrifugation (4000 g, 5 min) and were subsequently washed with sterile Artificial Sea Water (ASW, composition per liter: NaCl 23.926 g, Na_2_SO_4_ 4.008 g, KC1 0.677 g, NaHCO_3_ 0.196 g, KBr 0.098 g, H_3_BO_3_ 0.026 g and NaF 0.003 g). After autoclaving, cyanocobalamin (0.5 mg/L) and cycloheximide (25 mg/L) were added. Cells were resuspended in 4 mL of ASW and 50 µL of this solution were transferred as circular patches onto ASW agar plates, allowing them to dry for 2 h. A small amount of environmental sample was placed on the middle of each *E. coli* or *P. inhibens* patch. The following incubation was performed at 30 °C for 3 weeks. Once, lysis of *E. coli* or *P. inhibens* cells was observed, the agar piece of the lytic zone was cut out and transferred onto a new ASW agar plate. The same procedure was repeated, until an axenic culture was obtained.

### 4.2. Metagenomic Analysis of 16S rRNA Gene Sequence

For metagenomic analysis, 30 subsamples were collected at each sample site (100 m^2^ diameter). From each location the respective sample (1 g wet weight) was mixed with 5 mL deionized water and homogenized using a Potter homogenizator. The resulting mixture was filtered using a 5 µm pore size filter (Sartorius, Göttingen, Germany) to enrich bacterial cells. The cells were collected by centrifugation at 10,000 *g* for 15 min. The metagenomics DNA was isolated using the GenElute Bacterial Genomic DNA Kit (Sigma, Darmstadt, Germany) and the 16S rRNA gene was sequenced by CEMET GMBH (Tübingen, Germany) using an Illumina sequencer and F515/R806 primers. The obtained data was analyzed using Megan6 [40].

### 4.3. Antimicrobial Activity

To determine the antimicrobial activity of the isolated strains, a small-scale cultivation (50 mL) was carried out in marine broth M2216 (Difco, Sparks, MD, USA) for 4 days at 30 °C. Then, the cultures were extracted 1:1 with ethyl acetate and the organic phase was evaporated to dryness in a rotary evaporator. The resulting crude extract was resuspended in methanol to a final concentration of 10 mg/mL. 50 µL of these extracts were added to sterile filter disks and were placed onto LB agar plates on which the test organisms (*Arthrobacter psychrolactophilus* as Gram-positive and *Escherichia coli* XL1 Blue as Gram-negative) were previously streaked out. The plates were incubated overnight at 30° and 37 °C, respectively. The inhibition of the test strains was quantified by the diameter of the resulting halo (in mm). In the first screening round, 35 strains showed activity and were used for a further antimicrobial activity test against relevant pathogenic strains, i.e., methicillin resistant *Staphylococcus aureus* (MRSA) and enterohemorrhagic *E. coli* (EHEC).

### 4.4. Growth Characteristics

For the strains that showed antimicrobial activity, the temperature and salinity tolerance was determined. The growth response was investigated by testing several temperatures, ranging from 25 to 45 °C in marine broth agar M2216 (Difco, Sparks, MD, USA). The salinity tolerance was assessed by modifying the NaCl level of the medium from 0.5 to 10%.

### 4.5. Phylogenetic Analysis of Isolated Strains

The strains that showed antimicrobial activity against any of the test strains were selected for 16S rRNA identification. The genomic DNA of the respective strain was isolated using the DNA purification kit according to Promega manufacturer’s instructions. PCR amplification of the 16S rRNA gene was achieved using the primer pair pA (5′-AGAGTTTGATCCTGGCTCAG-3′) and pH (5′-AAGGAGGTGATCCAGCCCCA-3′). The resulting DNA fragments were gel purified and then sequenced (GATC, Konstanz, Germany) using the same primers. The data resulting from both sides of the end sequencing were merged and analyzed using BLASTn. The phylogenetic tree was built using the minimum evolution method of the software MEGA 7.

### 4.6. HPLC-MS/MS Measurements

The previously obtained crude extracts (see Section 2.3) were diluted in methanol to a final concentration of 1 mg/mL. Mass spectra were recorded on a micrOTOF-Q mass spectrometer (Bruker, Billerica, MA, USA) with ESI-source coupled with a HPLC Dionex Ultimate 3000 (Thermo Scientific, Darmstadt, Germany) using an EC10/2 Nucleoshell C18 2.7 µm column (Macherey-Nagel, Düren, Germany). The column temperature was 25 °C. MS data were acquired over a range from 100 to 3000 *m*/*z* in positive mode. Auto MS/MS fragmentation was achieved with rising collision energy (35–50 keV over a gradient from 500 to 2000 *m*/*z*) with a frequency of 4 Hz for all of the ions over a threshold of 100. HPLC begins with 90% H_2_O containing 0.1% acetic acid. The gradient starts after 1 min to 100% acetonitrile (0.1% acetic acid) in 20 min. 5 µL of a 1 mg/mL sample solution was injected; and, flow rate was set to 0.3 mL/min.

## Figures and Tables

**Table 1:** Number of reads of different reported predatory taxa in the metagenomic analysis of 16S rRNA gene sequences.

Taxa	Site of Collection (N° of Reads) ^a^		
	Isla Foca (213,991)	Paracas (161,958)	Manglares (163,056)
**Bacteroidetes**			
Cytophagales	6	203	11
Cellulophaga	2	0	0
Tenacibaculum	4	131	179
Saprospiraceae	2	334	0
**Delta-proteobacteria**			
Bdellovibrionales-like bacteria	2	81	1184
Bacteriovoracaceae	1	324	2
Bdellovibrionaceae	2	3	0
Myxococcales	0	1	0
**Cyanobacteria**			
Vampirovibrio	0	0	2

^a^ The total number of reads obtained by Illumina sequencing is given.

## Supplementary Materials

The following are available online at www.mdpi.com/1660-3397/15/10/308/s1, Table S1: Isolated strains that showed antibiotic activity, Table S2: Growth behavior at different NaCl concentrations, Table S3: Growth behavior at different temperatures, Table S4: Selected predatory bacteria reported in the literature. The 16S rRNA sequences of the strains have been deposited at GenBank with the accession numbers MF796603-MF796631 and MF620093, Table S4: Antismash bioinformatic of secondary metabolites BGC present in the genome of *Streptomyces* sp. s120 showing the presence of the naphthyridinomycin and resistomycin BGC.
