# Supplementary material for: Diversity and Antimicrobial Potential of Predatory Bacteria from the Peruvian Coastline
Source: Mar Drugs. 2017 Oct 12;15(10):308. doi: 10.3390/md15100308 (PMC5666416; doi:10.3390/md15100308)
Supplement: Supplementary file 1 [file marinedrugs-15-00308-s001.pdf]

Supplementary Material

Diversity and Antimicrobial Potential of Predatory Bacteria from the Peruvian Coastline

Luis Linares-Otoya,[1,2,5] Virginia Linares-Otoya,[3,5] Lizbeth Armas-Mantilla,[3] Cyntia Blanco-Olano,[3] Max Crüsemann,[2] Mayar L. Ganoza-Yupanqui,[3] Julio Campos-Florian,[3] Gabriele M. König,[2,4] Till F. Schäberle[1,2,4,*]

[1] Institute for Insect Biotechnology, Justus Liebig University of Giessen, Giessen, Germany
[2] Institute for Pharmaceutical Biology, University of Bonn, Bonn, Germany
[3] Department of Pharmacology, Faculty of Pharmacy and Biochemistry, National University of Trujillo, Trujillo, Peru
[4] German Centre for Infection Research (DZIF) Partner Site Bonn/Cologne
[5] Research Centre for Sustainable Development Uku Pacha, Peru
* Correspondence till.f.schaeberle@agrar.uni-giessen.de; Tel.: +49-641-9937140

Supplementary Table S1. Isolated strains that showed antibiotic activity

Strain identifier	Strain name as deposited in NCBI database	Closest 16S rRNA gene sequence hit in BLAST	Identity %	Isolation bait	Isolation site
2	Tenacibacullum sp. s2	Tenacibacullum sp. QDHT-02	99	*E. coli*	Paracas
16	Tenacibaculum sp. S16	Tenacibaculum sp. sw0106-03(3)	99	*E. coli*	Paracas
17	Kocuria rosea s17	Kocuria rosea	99	*E. coli*	Paracas
21	Reichenbachiella sp. S21	Reichenbachiella agariperforans	95	*E. coli*	Paracas
23	Oceanicola marinus s23	Oceanicola marinus Sl5	99	*E. coli*	Paracas
37	Limibacter armeniacum s37	Limibacter armeniacum YM11-159	99	*E. coli*	Paracas
42	Fulvivirga sp. s42	Fulvivirga sp. ana H.e.	99	*P. inhibens*	Isla Foca
47	Porifericola rodea s47	Porifericola rhodea	99	*P. inhibens*	Paracas
48	Fulvivirga kasyanovii s48	Fulvivirga kasyanovii	99	*P. inhibens*	Isla Foca
68	Rapidithrix sp. s68	Rapidithrix sp. TISTR 1768	99	*P. inhibens*	Paracas
80	Rapidithrix sp s80	Rapidithrix sp. TISTR 1768	99	*P. inhibens*	Paracas
82	Labrenzia sp. strain s82	Labrenzia sp. strain 1334-392	98	*E. coli*	Paracas
83	Microbulbifer hydrolyticus s83	Microbulbifer hydrolyticus strain DSM 11525	99	*E. coli*	Manglares
90	Bacillus sp. s90	Bacillus thuringiensis serovar finitimus YBT-020	99	*P. inhibens*	Paracas
93	Labrenzia sp. s93	Labrenzia sp. R-66638	99	*E. coli*	Isla Foca
100	Nitratireductor sp. s100	Nitratireductor sp.	99	*E. coli*	Manglares
106	Staphylococcus succinus s106	Staphylococcus succinus	99	*P. inhibens*	Paracas
107	Microbulbifer sp. s107	Microbulbifer sp. CMC	99	*P. inhibens*	Manglares
108	Euzebyella sp. s108	Euzebyella sp. CY01	97	*P. inhibens*	Manglares
120	Streptomyces sp. s120	Streptomyces sp. NPA1	99	*E. coli*	Manglares
122	Nocardiopsis sp. s122	Nocardiopsis sp. 13-112	99	*E. coli*	Manglares
123	Nitratireductor sp. s123	Nitratireductor sp. OM-1	99	*E. coli*	Isla Foca
102[a]	Microbacterium sp. s102a	Microbacterium sp. CC4P	99	*P. inhibens*	Paracas
102b	Paenibacillus sp. s102b	Paenibacillus glucanolyticus strain 5162	99	*P. inhibens*	Paracas
118b	Echinicola shivajiensis s118b	Echinicola shivajiensis strain AK12	99	*E. coli*	Manglares
17_1c	Planomicrobium sp. s171c	Planomicrobium sp. PK32_S1	99	*E. coli*	Paracas
17_2c	Paracoccus sp. s172c	Paracoccus sp. MAR824	98	*E. coli*	Paracas
30b1	Ponticoccus sp. s30b1	Ponticoccus sp. strain 7002-260	100	*E. coli*	Paracas
49b1	Bacillus aquimaris s49b1	Bacillus aquimaris strain M12	99	*E. coli*	Paracas
49b2	Euzebyella sp. s49b2	Euzebyella sp. B39	99	*E. coli*	Paracas

Supplementary Table S2. Growth behavior at different NaCl concentrations

Strain identifier	% NaCl							
	0.5%	1%	1.95%	2.14%	2.23%	3.89%	5%	10%
2	--	++	+++	+++	++	++	--	--
16	--	+	++	+++	++	+	+	--
17	++	++	++	++	++	++	+	--
17_1c	++	++	++	++	++	++	+	--
17_2c	++	++	++	++	++	++	+	--
21	+	+	++	+++	++	++	+	+
23	--	--	--	+	++	+++	++	--
30b1	++	+++	+++	++++	+++	+++	+++	++
37	++	++	++	++	++	++	--	--
42	+	++	+++	+++	++	+	+	+
47	+	++	+++	++	++	++	--	--
48	+	++	++	++	+	--	--	--
49b1	+++	+++	+++	+++	+++	++	+	--
49b2	+	+	++	++	++	++	+	--
68	+	++	+++	+++	++	+	--	--
80	+	++	+++	+++	++	+	--	--
82	++	++	+++	+++	++	++	++	--
83	+	++	+++	+++	++	++	++	+
90	+++	+++	+++	+++	+++	+++	++	--
93	+	++	++	++	+	+	--	--
100	++	++	++	++	++	++	++	-
102a	+	++	++	++	++	+	+	+
102b	+	++	+++	+++	++	+	+	+
106	++	+++	++++	+++	+++	+++	++	++
107	+	++	+++	+++	++	+	+	+
108	+	+	++	+++	++	+	+	--
118b	--	+	++	++	+	+	+	--
120	++	++	++	++	++	++	--	--
122	+	++	++	++	++	+	+	+
123	++	+++	+++	++	++	++	+	+

Supplementary Table S3. Growth behavior at different temperatures

Strain identifier	10ºC		27 ºC		30ºC		45ºC	
	24 H	48 H	24 H	48 H	24 H	48 H	24 H	48 H
2	--	--	++	+++	++	+++	--	--
16	--	--	++	+++	++	+++	--	--
17	--	--	++	++	++	++	--	--
17_1c	--	--	++	++	++	++	+	+
17_2c	--	--	++	++	++	++	--	--
21	--	--	++	+++	++	+++	--	--
23	--	--	++	+++	++	+++	--	--
30b1	--	--	+	++	++	+++	--	--
37	--	--	++	++	++	+++	--	--
42	--	--	--	+	+	++	--	--
47	--	--	--	+	++	+++	+	+
48	--	--	--	+	+	++	--	--
49b1	--	--	+	++	+	++	+	+
49b2	--	--	--	++	++	+++	--	--
68	--	--	--	++	+	++	--	--
80	--	--	+++	+++	+++	+++	--	--
80	--	--	--	++	+	++	--	--
82	--	--	++	+++	++	+++	+	+
83	--	--	+	++	++	+++	--	+
90	--	--	++	+++	++	+++	++	++
93	--	--	+	++	+	++	+	+
100	--	--	++	++	++	++	--	--
102a	--	--	+	++	+	++	+	+
102b	--	--	+	++	+	++	+	+
106	--	--	++	++	++	+++	+++	+++
107	--	--	+	++	++	+++	+	+
108	--	--	++	+++	+++	+++	--	--
118b	--	--	--	+	+	++	+	+
120	--	--	++	+++	+++	+++	++	+++
122	--	--	+	++	+	++	+	+
123	--	--	+	++	+	++	+	+

Supplementary Table S4. Selected predatory bacteria reported in the literature

**Genus and or species**	**Reference**
Agromyces ramosus	[1]
Herpetosiphon	[2]
Bdellovibrio bacteriovorus	[3]
Halobacteriovorax	[4]
Myxococcus xanthus	[2]
Vampirococcus	[5]
Ensifer	[6]
Cupriavidus necator	[7]
Lysobacter	[8]
Saprospira grandis	[9]
Tenacibaculum	[10]
Olleya	[10]
Cytophaga	[11]
Rapidithrix	[12]
Poriferícola rhodea	[13]
Vampirovibrio chlorellavorus	[2]
Micavibrio	[14]

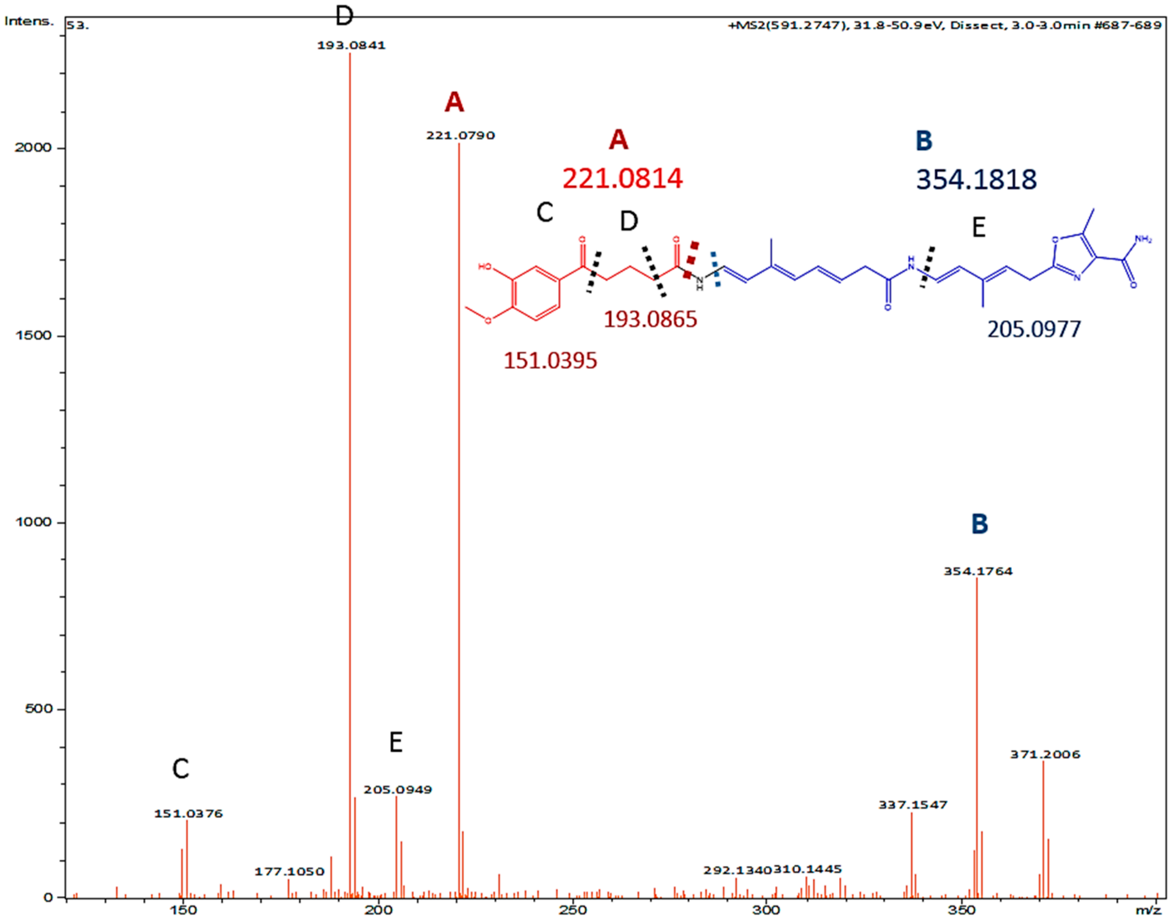

Supplementary Figure S1. MS/MS spectrum of *Rapidithrix thailandica* s80 crude extract showing the fragmentation pattern expected for ariakemicins isolated before from *Rapidithrix* sp [15].

Supplementary Table S5. Antismash bioinformatic of secondary metabolites BGC present in the genome of *Streptomyces sp*. s120 showing the presence of the naphthyridinomycin and resistomycin BGC [16].

Type	From	To	Most similar known cluster	MIBiG BGC-ID
Otherks-T1pks-Nrps	1	58334	Naphthyridinomycin_biosynthetic_gene_cluster (60% of genes show similarity)	BGC0000394_c1
Other	1	16023	Naphthyridinomycin_biosynthetic_gene_cluster (39% of genes show similarity)	BGC0000394_c1
T2pks	422	42943	Resistomycin_biosynthetic_gene_cluster (88% of genes show similarity)	BGC0000264_c1

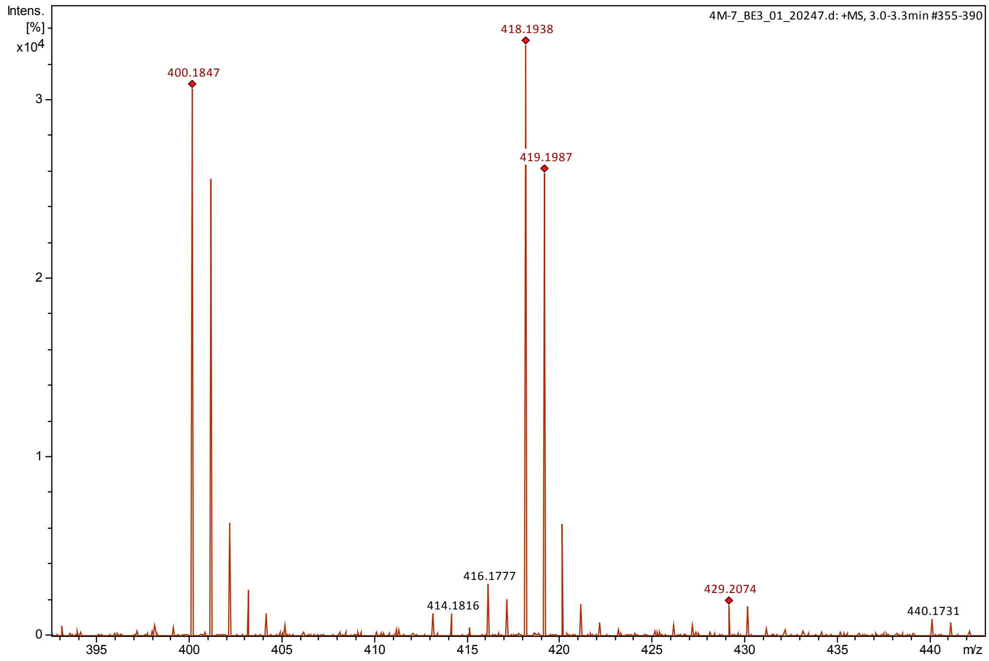

Supplementary Figure S2. MS spectrum of *Streptomyces sp*. s120 extract showing the presence of naphthyridinomycin (observed *m/z* 418.1938, expected 418.1978) [17].

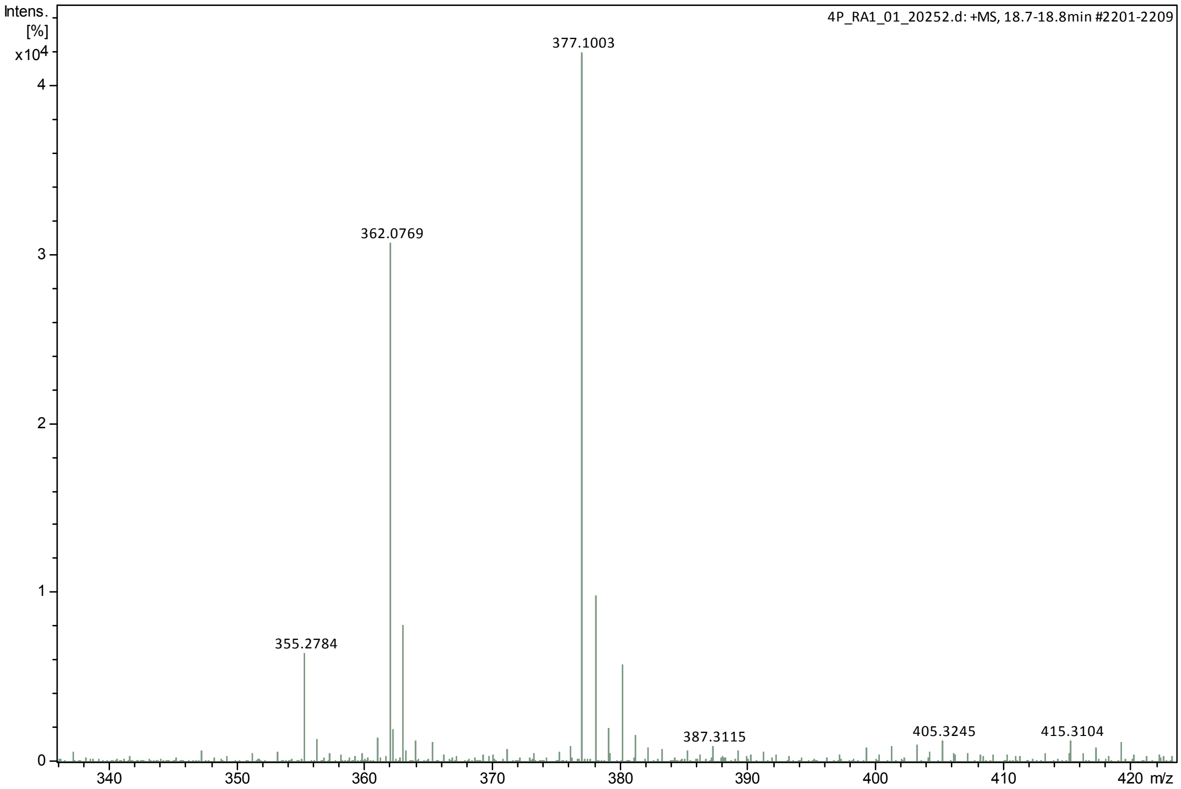

Supplementary Figure S3. MS spectrum of *Streptomyces sp*. s120 extract showing the presence of resistomycin (observed *m/z* 377.1003, expected 377.1025) [18].

Supplementary Table S6. GNPS dereplication analysis of crude extract of *Paenibacillus sp*. s102b showing the presence of pumilacidin C and E as well as surfactin [19].

Compound_Name	Library Class	Cosine	Shared Peaks	TIC	RT	Mass Diff	SpecMZ	LibMZ	PI
pumilacidin_C_14344_dereplictor_pv_2.93242e-26	Bronze	0.82	36	6691.19	419.89	0.01	1078.75	1078.74	Pevzner
pumilacidin_E_14342_dereplictor_pv_1.13518e-25	Bronze	0.65	25	4486.22	402.57	0.02	1064.73	1064.72	Pevzner
[Ile2_4_7]Surfactin_43293_dereplictor_pv_2.3158e-16	Bronze	0.73	20	1797.96	1152.71	0.01	1022.68	1022.67	Pevzner

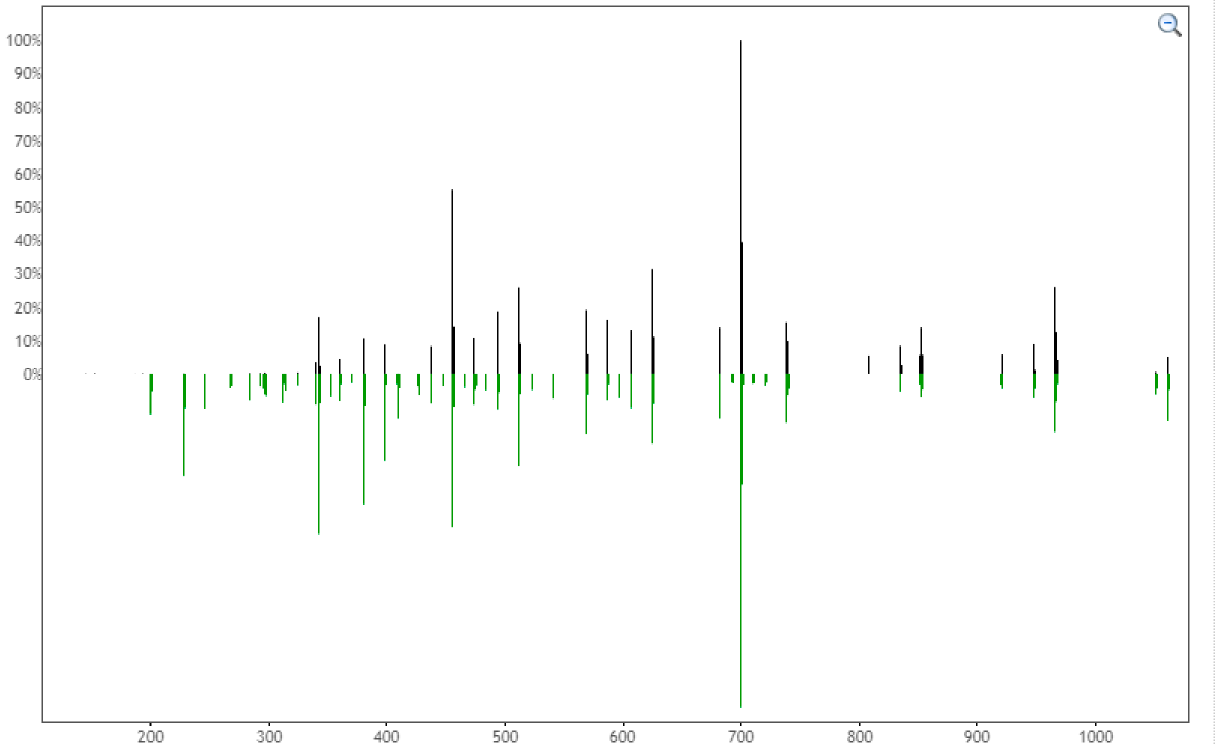

Supplementary Figure S4. MS/MS mirror plot of crude extract of *Paenibacillus sp*. s102b against GNPS database showing the presence of pumilacidin C [19]. Source: Green: Pumilacidin C MS/MS spectrum from GNPS database, black: Crude extract of *Paenibacillus sp*. s102b.

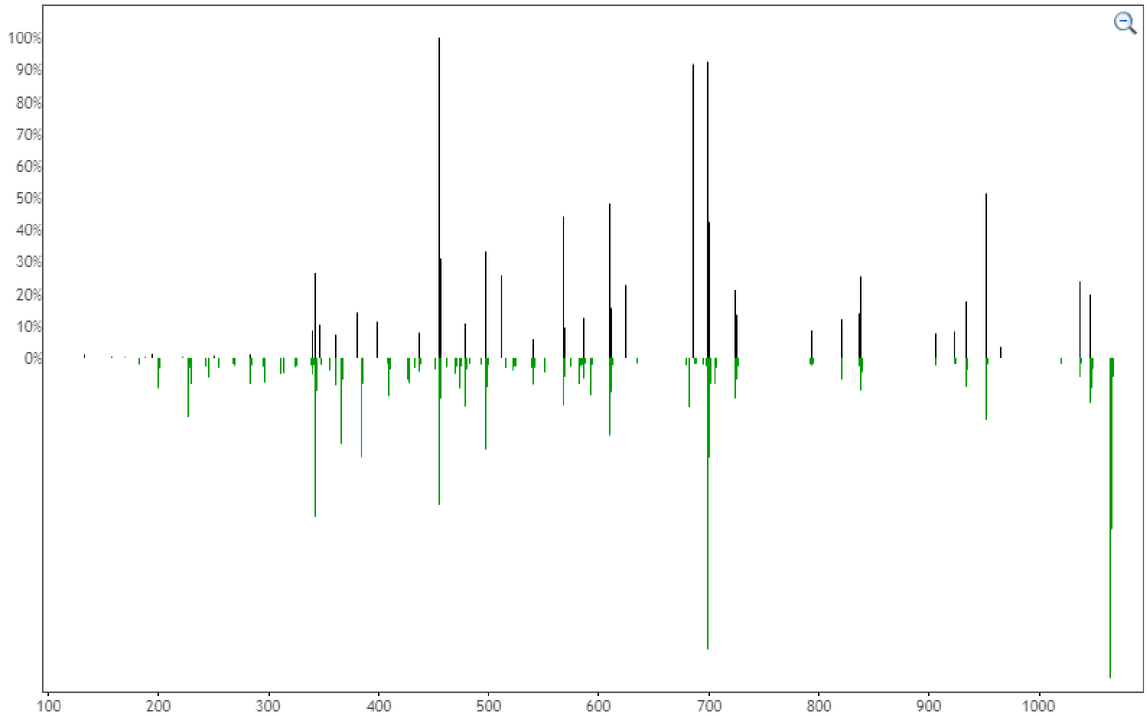

Supplementary Figure S5. MS/MS mirror plot of crude extract of *Paenibacillus sp*. s102b against GNPS database showing the presence of pumilacidin E [19]. Source: Green: Pumilacidin E MS/MS spectrum from GNPS database Black: Crude extract of *Paenibacillus sp*. s102b.

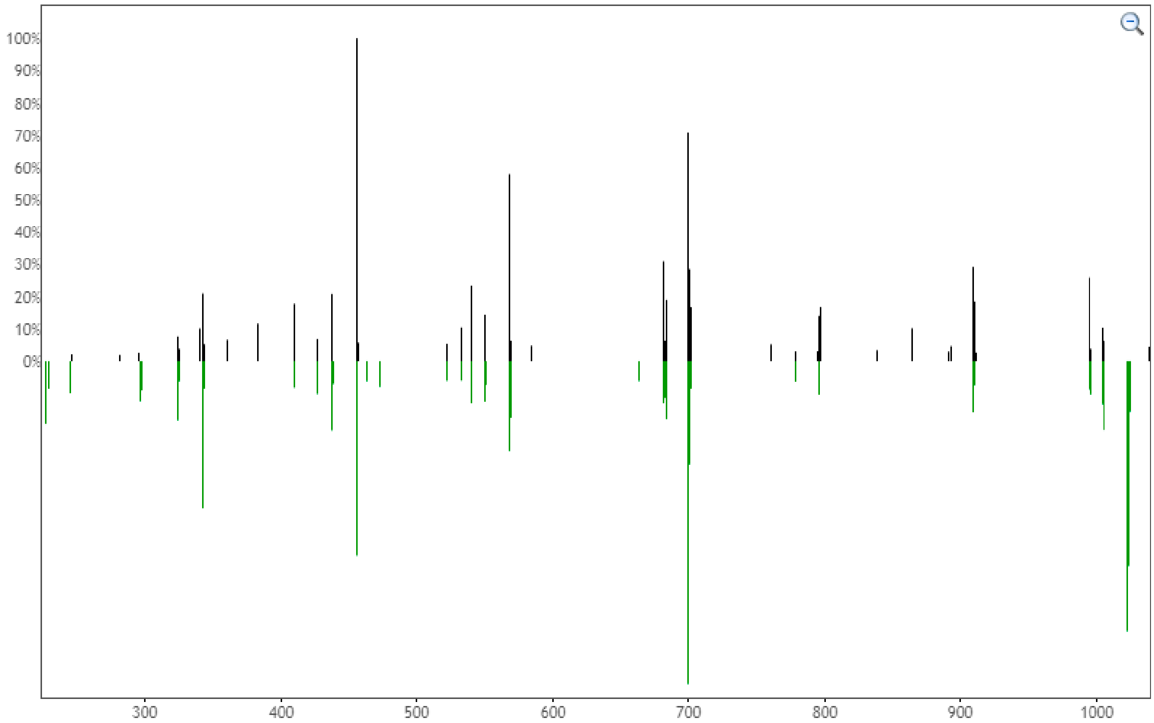

Supplementary Figure S6. MS/MS mirror plot of crude extract of *Paenibacillus sp*. s102b against GNPS database showing the presence of surfactin [19]. Source: Green: Surfactin MS/MS spectrum from GNPS database, black: Crude extract of *Paenibacillus sp*. s102b.

References

1. Casida, L.E. Interaction of Agromyces ramosus with Other Bacteria in Soil. Applied And Environmental Microbiology 1983, 46, 881–888.

2. Korp, J.; Vela Gurovic, M.S.; Nett, M. Antibiotics from predatory bacteria. Beilstein journal of organic chemistry 2016, 12, 594–607.

3. Jurkevitch, E.; Jacquet, S. Bdellovibrio and related organisms Unusual bacterial predators! M S-Medecine Sciences 2017, 33, 519–527.

4. Kandel, P.P.; Pasternak, Z.; van Rijn, J.; Nahum, O.; Jurkevitch, E. Abundance, diversity and seasonal dynamics of predatory bacteria in aquaculture zero discharge systems. FEMS microbiology ecology 2014, 89, 149–161.

5. Guerrero, R.; Pedrosalio, C.; Esteve, I.; Mas, J.; Chase, D.; Margulis, L. Predatory prokaryotes - predation and primary consumption evolved in bacteria. Proceedings of the National Academy of Sciences of the United States of America 1986, 83, 2138–2142.

6. Willems, A.; Fernández-López, M.; Muñoz-Adelantado, E.; Goris, J.; Vos, P. de; Martínez-Romero, E.; Toro, N.; Gillis, M. Description of new Ensifer strains from nodules and proposal to transfer Ensifer adhaerens Casida 1982 to Sinorhizobium as Sinorhizobium adhaerens comb. nov. Request for an opinion. International Journal Of Systematic And Evolutionary Microbiology 2003, 53, 1207–1217.

7. Seccareccia, I.; Kovacs, A.T.; Gallegos-Monterrosa, R.; Nett, M. Unraveling the predator-prey relationship of Cupriavidus necator and Bacillus subtilis. Microbiological Research 2016, 192, 231–238.

8. Seccareccia, I.; Kost, C.; Nett, M. Quantitative Analysis of Lysobacter Predation. Applied And Environmental Microbiology 2015, 81, 7098–7105.

9. Lewin, R.A. Saprospira grandis: A flexibacterium that can catch bacterial prey by "ixotrophy". Microbial Ecology 1997, 34, 232–236.

10. Banning, E.C.; Casciotti, K.L.; Kujawinski, E.B. Novel strains isolated from a coastal aquifer suggest a predatory role for flavobacteria. FEMS microbiology ecology 2010, 73, 254–270.

11. Imai, I.; Ishida, Y.; Hata, Y. Killing of marine-phytoplankton by a gliding bacterium Cytophaga sp, isolated from the coastal sea of Japan. Marine Biology 1993, 116, 527–532.

12. Srisukchayakul, P.; Suwanachart, C.; Sangnoi, Y.; Kanjana-Opas, A.; Hosoya, S.; Yokota, A.; Arunpairojana, V. Rapidithrix thailandica gen. nov., sp nov., a marine gliding bacterium isolated from samples collected from the Andaman sea, along the southern coastline of Thailand. International Journal Of Systematic And Evolutionary Microbiology 2007, 57, 2275–2279.

13. Yoon, J.; Oku, N.; Park, S.; Kasai, H.; Yokota, A. Porifericola rhodea gen. nov., sp nov., a new member of the phylum Bacteroidetes isolated by the bait-streaked agar technique. Antonie Van Leeuwenhoek International Journal Of General And Molecular Microbiology 2011, 100, 145–153.

14. Dashiff, A.; Junka, R.A.; Libera, M.; Kadouri, D.E. Predation of human pathogens by the predatory bacteria Micavibrio aeruginosavorus and Bdellovibrio bacteriovorus. Journal of applied microbiology 2011, 110, 431–444.

15. Oku, N.; Adachi, K.; Matsuda, S.; Kasai, H.; Takatsuki, A.; Shizuri, Y. Ariakemicins a and b, novel polyketide-peptide antibiotics from a marine gliding bacterium of the genus Rapidithrix. Organic Letters 2008, 10, 2481–2484.

16. Blin, K.; Wolf, T.; Chevrette, M.G.; Lu, X.; Schwalen, C.J.; Kautsar, S.A.; Suarez Duran, H.G.; Los Santos, E.L.C. de; Kim, H.U.; Nave, M.; et al. antiSMASH 4.0-improvements in chemistry prediction and gene cluster boundary identification. Nucleic acids research 2017.
17. Zmijewski, M.J.; Millerhatch, K.; Goebel, M. Naphthyridinomycin, a DNA-reactive antibiotic. Antimicrobial agents and chemotherapy 1982, 21, 787–793.
18. Rosenbrook, W. The structure of resistomycin. The Journal of organic chemistry 1967, 32, 2924–2925.
19. Wang, M.; Carver, J.J.; Phelan, V.V.; Sanchez, L.M.; Garg, N.; Peng, Y.; Nguyen, D.D.; Watrous, J.; Kapono, C.A.; Luzzatto-Knaan, T.; et al. Sharing and community curation of mass spectrometry data with Global Natural Products Social Molecular Networking. Nature Biotechnology 2016, 34, 828–837.

References

1. Casida, L.E. Interaction of Agromyces ramosus with Other Bacteria in Soil. *APPLIED AND ENVIRONMENTAL MICROBIOLOGY* **1983**, *46*, 881–888.
2. Korp, J.; Vela Gurovic, M.S.; Nett, M. Antibiotics from predatory bacteria. *Beilstein journal of organic chemistry* **2016**, *12*, 594–607.
3. Jurkevitch, E.; Jacquet, S. Bdellovibrio and related organisms Unusual bacterial predators! *M S-MEDECINE SCIENCES* **2017**, *33*, 519–527.
4. Kandel, P.P.; Pasternak, Z.; van Rijn, J.; Nahum, O.; Jurkevitch, E. Abundance, diversity and seasonal dynamics of predatory bacteria in aquaculture zero discharge systems. *FEMS microbiology ecology* **2014**, *89*, 149–161.
5. GUERRERO, R.; PEDROSALIO, C.; ESTEVE, I.; MAS, J.; CHASE, D.; MARGULIS, L. PREDATORY PROKARYOTES - PREDATION AND PRIMARY CONSUMPTION EVOLVED IN BACTERIA. *Proceedings of the National Academy of Sciences of the United States of America* **1986**, *83*, 2138–2142.
6. Willems, A.; Fernández-López, M.; Muñoz-Adelantado, E.; Goris, J.; Vos, P. de; Martínez-Romero, E.; Toro, N.; Gillis, M. Description of new Ensifer strains from nodules and proposal to transfer Ensifer adhaerens Casida 1982 to Sinorhizobium as Sinorhizobium adhaerens comb. nov. Request for an opinion. *INTERNATIONAL JOURNAL OF SYSTEMATIC AND EVOLUTIONARY MICROBIOLOGY* **2003**, *53*, 1207–1217.
7. Seccareccia, I.; Kovacs, A.T.; Gallegos-Monterrosa, R.; Nett, M. Unraveling the predator-prey relationship of Cupriavidus necator and Bacillus subtilis. *MICROBIOLOGICAL RESEARCH* **2016**, *192*, 231–238.
8. Seccareccia, I.; Kost, C.; Nett, M. Quantitative Analysis of Lysobacter Predation. *APPLIED AND ENVIRONMENTAL MICROBIOLOGY* **2015**, *81*, 7098–7105.
9. Lewin, R.A. Saprospira grandis: A flexibacterium that can catch bacterial prey by ''ixotrophy''. *MICROBIAL ECOLOGY* **1997**, *34*, 232–236.
10. Banning, E.C.; Casciotti, K.L.; Kujawinski, E.B. Novel strains isolated from a coastal aquifer suggest a predatory role for flavobacteria. *FEMS microbiology ecology* **2010**, *73*, 254–270.
11. IMAI, I.; ISHIDA, Y.; HATA, Y. KILLING OF MARINE-PHYTOPLANKTON BY A GLIDING BACTERIUM CYTOPHAGA SP, ISOLATED FROM THE COASTAL SEA OF JAPAN. *MARINE BIOLOGY* **1993**, *116*, 527–532.

12. Srisukchayakul, P.; Suwanachart, C.; Sangnoi, Y.; Kanjana-Opas, A.; Hosoya, S.; Yokota, A.; Arunpairojana, V. Rapidithrix thailandica gen. nov., sp nov., a marine gliding bacterium isolated from samples collected from the Andaman sea, along the southern coastline of Thailand. *INTERNATIONAL JOURNAL OF SYSTEMATIC AND EVOLUTIONARY MICROBIOLOGY* **2007**, *57*, 2275–2279.
13. Yoon, J.; Oku, N.; Park, S.; Kasai, H.; Yokota, A. Porifericola rhodea gen. nov., sp nov., a new member of the phylum Bacteroidetes isolated by the bait-streaked agar technique. *ANTONIE VAN LEEUWENHOEK INTERNATIONAL JOURNAL OF GENERAL AND MOLECULAR MICROBIOLOGY* **2011**, *100*, 145–153.
14. Dashiff, A.; Junka, R.A.; Libera, M.; Kadouri, D.E. Predation of human pathogens by the predatory bacteria Micavibrio aeruginosavorus and Bdellovibrio bacteriovorus. *Journal of applied microbiology* **2011**, *110*, 431–444.
15. Oku, N.; Adachi, K.; Matsuda, S.; Kasai, H.; Takatsuki, A.; Shizuri, Y. Ariakemicins a and b, novel polyketide-peptide antibiotics from a marine gliding bacterium of the genus Rapidithrix. *ORGANIC LETTERS* **2008**, *10*, 2481–2484.
16. Blin, K.; Wolf, T.; Chevrette, M.G.; Lu, X.; Schwalen, C.J.; Kautsar, S.A.; Suarez Duran, H.G.; Los Santos, E.L.C. de; Kim, H.U.; Nave, M.*; et al.* antiSMASH 4.0-improvements in chemistry prediction and gene cluster boundary identification. *Nucleic acids research* **2017**.
17. ZMIJEWSKI, M.J.; MILLERHATCH, K.; GOEBEL, M. NAPHTHYRIDINOMYCIN, A DNA-REACTIVE ANTIBIOTIC. *Antimicrobial agents and chemotherapy* **1982**, *21*, 787–793.
18. Rosenbrook, W. The structure of resistomycin. *The Journal of organic chemistry* **1967**, *32*, 2924–2925.
19. Wang, M.; Carver, J.J.; Phelan, V.V.; Sanchez, L.M.; Garg, N.; Peng, Y.; Nguyen, D.D.; Watrous, J.; Kapono, C.A.; Luzzatto-Knaan, T.*; et al.* Sharing and community curation of mass spectrometry data with Global Natural Products Social Molecular Networking. *NATURE BIOTECHNOLOGY* **2016**, *34*, 828–837.
